# Combating Pathogenic Microorganisms Using Plant-Derived Antimicrobials: A Minireview of the Mechanistic Basis

**DOI:** 10.1155/2014/761741

**Published:** 2014-09-14

**Authors:** Abhinav Upadhyay, Indu Upadhyaya, Anup Kollanoor-Johny, Kumar Venkitanarayanan

**Affiliations:** Department of Animal Science, University of Connecticut, 3636 Horsebarn Hill Road Extension, Unit 4040, Storrs, CT 06269, USA

## Abstract

The emergence of antibiotic resistance in pathogenic bacteria has led to renewed interest in exploring the potential of plant-derived antimicrobials (PDAs) as an alternative therapeutic strategy to combat microbial infections. Historically, plant extracts have been used as a safe, effective, and natural remedy for ailments and diseases in traditional medicine. Extensive research in the last two decades has identified a plethora of PDAs with a wide spectrum of activity against a variety of fungal and bacterial pathogens causing infections in humans and animals. Active components of many plant extracts have been characterized and are commercially available; however, research delineating the mechanistic basis of their antimicrobial action is scanty. This review highlights the potential of various plant-derived compounds to control pathogenic bacteria, especially the diverse effects exerted by plant compounds on various virulence factors that are critical for pathogenicity inside the host. In addition, the potential effect of PDAs on gut microbiota is discussed.

## 1. Introduction

Human population growth with its global effects on the environment over the past million years has resulted in the emergence of infectious diseases [1, 2]. Development of agriculture further contributed to this, since these infections could only be sustained in large and dense human populations [3]. The discovery of antibiotics during the twentieth century coupled with significant advances in antimicrobial drug development improved human health through improved treatment of infections [4, 5]. However, prolonged use of antibiotics led to bacterial adaptation, resulting in the development of multidrug resistance in bacteria [2, 5–8]. This has significantly limited the efficacy of antibiotics, warranting alternative strategies to combat microbial infections.

The persistence of bacteria in the environment and their interaction with humans is central to most infections and illnesses. Bacterial illnesses are orchestrated by means of an array of virulence factors that facilitate various aspects of their pathophysiology critical for disease in the host [9]. These include adhesins and membrane proteins that mediate bacterial attachment, colonization, and invasion of host cells. In addition, microbial toxins cause host tissue damage, and bacterial cell wall components such as capsular polysaccharide confer resistance against host immune system [10, 11]. Biofilm formation and spore forming capacity are additional virulence factors that help in the persistence of pathogens in harsh environmental conditions.

Since ancient times, plants have played a critical role in the development and well-being of human civilization. A plethora of plant products have been used as food preservatives, flavor enhancers, and dietary supplements to prevent food spoilage and maintain human health. In addition, plant extracts have been widely used in herbal medicine, both prophylactically and therapeutically for controlling diseases. The antimicrobial activity of several plant-derived compounds has been previously reported [12–23], and a wide array of active components have been identified [24]. A majority of these compounds are secondary metabolites and are produced as a result of reciprocal interactions between plants, microbes, and animals [25]. These compounds do not appear to play a direct role in plant physiology [26]; however they are critical for enhancing plant fitness and defense against predation [27]. The production of secondary metabolites is often restricted to a limited set of species within a phylogenetic group as compared to primary metabolites (amino acids, polysaccharides, proteins, and lipids), which are widespread in the plant kingdom [28]. Also, they are generated only during a specific developmental period of plant growth at micro- to submicromolar concentration [28, 29].

The primary advantage of using plant-derived antimicrobials (PDAs) for therapeutic purposes is that they do not exhibit the side effects often associated with use of synthetic chemicals [30]. In addition, to the best of our knowledge, no reports of antimicrobial resistance to these phytochemicals have been documented, probably due to their multiple mechanisms of action which potentially prevent the selection of resistant strains of bacteria. The marked antimicrobial effect, nontoxic nature, and affordability of these compounds have formed the basis for their wide use as growth promoters in the livestock and poultry industry, effective antimicrobials and disinfectants in the food industry, components of herbal therapy in veterinary medicine, and source for development of novel antibiotics in pharmaceutics.

The antimicrobial properties of various plant compounds that target cellular viability of bacteria have been adequately discussed previously [12, 31–33], but very few reviews have highlighted the effects of these compounds in modulating various aspects of bacterial virulence, critical for pathogenesis in the host. In this review, we have focused on a wide array of PDAs, with special emphasis on the diverse biological effects exerted by these compounds on bacterial virulence. The important classes of plant compounds and selected antimicrobial mechanisms have been discussed.

## 2. Plant-Derived Antimicrobials

Most plant-derived compounds are produced as secondary metabolites and can be classified based on their chemical structure, which also influences their antimicrobial property (Table 1). The major groups of phytochemicals are presented here.

### 2.1. Phenolics and Polyphenols

These are a diverse group of aromatic secondary metabolites involved in plant defense. They consist of flavonoids, quinones, tannins, and coumarins [33–35].

#### 2.1.1. Flavonoids

Flavonoids are pigmented compounds found in fruits and flowers of plants and mainly consist of flavone, flavanones, flavanols, and anthocyanidins [34, 35]. They facilitate pollination by acting as chemoattractants for insects, modulate plant physiology by signaling to beneficial microbiota in rhizosphere, and protect plants against predation due to their antimicrobial nature [36]. The marked antimicrobial property of flavonoids against a variety of bacterial [37–39] and fungal pathogens [40] is mediated by their action on the microbial cell membranes [41]. They interact with membrane proteins present on bacterial cell wall leading to increased membrane permeability and disruption. Catechins belonging to this group exhibit inhibitory activity against both Gram-positive and Gram-negative organisms [42].

#### 2.1.2. Quinones

Quinones are organic compounds consisting of aromatic rings with two ketone substitutions. Quinones are known to complex irreversibly with nucleophilic amino acids in protein, often leading to their inactivation and loss of function [43]. The major targets in the microbial cell include surface-exposed adhesin proteins, cell wall polypeptides, and membrane-bound enzymes [44]. Quinone such as anthraquinone from* Cassia italica* was found to be bacteriostatic against pathogenic bacteria such as* Bacillus anthracis*,* Corynebacterium pseudodiphthericum*, and* Pseudomonas aeruginosa* and bactericidal against* Burkholderia pseudomallei* [45].

#### 2.1.3. Tannins

Tannins are a group of water-soluble oligomeric and polymeric polyphenolic compounds, with significant astringent properties. They are present in the majority of plant parts, including bark, leave, fruits, and roots [46]. They are widely used in leather industry, in food industry, and, as antimicrobials, in healthcare industry [47]. The mode of antimicrobial action of tannins is potentially due to inactivation of microbial adhesins and cell envelope transport proteins [47–49]. Besides their efficacy against bacteria, tannins have been reported to be inhibitory on fungi and yeasts [46, 50].

#### 2.1.4. Coumarins

Coumarins are a group of aromatic benzopyrones consisting of fused benzene and alpha pyrone rings [51]. Approximately, 1300 coumarins have been identified since 1996 [44] and are used as antithrombotic and anti-inflammatory compounds [52]. Recently, coumarins such as scopoletin and chalcones have been isolated as antitubercular constituents of the plant* Fatoua pilosa* [53]. In addition, phytoalexins, which are hydroxylated derivatives of coumarins, which are produced in plants in response to microbial infections, have been found to exert marked antifungal activity.

### 2.2. Alkaloids

Alkaloids are a group of heterocyclic nitrogenous compounds with broad antimicrobial activity. Morphine and codeine are the oldest known compounds in this group [54]. Diterpenoid alkaloids, commonly isolated from Ranunculaceae or buttercup family of plants, are found to possess antimicrobial properties [55]. The mechanism of action of aromatic planar quaternary alkaloids such as berberine and harmane is attributed to their ability to intercalate with DNA thereby resulting in impaired cell division and cell death [33].

### 2.3. Terpenoids

Terpenes represent one of the largest and most diverse groups of secondary metabolites consisting of five carbon isoprene structural units linked in various configurations [43]. The action of terpene cyclase enzymes along with subsequent oxidation and structural rearrangement imparts a rich diversity to the group with over 55,000 members isolated so far [56]. The major groups consist of diterpenes, triterpenes, tetraterpenes as well as hemiterpenes, and sesquiterpenes [44]. When the compounds contain additional elements, frequently oxygen, they are termed terpenoids. Compounds such as menthol and camphor (monoterpenes), farnesol and artemisinin (sesquiterpenoids) are terpenoids synthesized from acetate units and share their origins and chemical properties with fatty acids [34]. Sesquiterpenoids are known to exhibit bactericidal activity against Gram-positive bacteria, including* M. tuberculosis* [35, 53]. The mechanism of antimicrobial action of terpenoids is not clearly defined, but it is attributed to membrane disruption in microorganisms [57].

### 2.4. Lectins and Polypeptides

In 1942, it was first reported that peptides could be inhibitory on microorganisms [58]. Although recent interest has chiefly focused on studying anti-HIV peptides and lectins, the inhibition of bacteria and fungi by these molecules has long been known [59]. The mechanism of action of peptides and lectins is presumed to be due to the formation of ion channels in the microbial membrane [60] or due to competitive inhibition of adhesion of microbial proteins to host polysaccharide receptors [61]. Lectin molecules are larger and include mannose-specific molecules obtained from an array of plants [62]. Lectins such as MAP30 from bitter melon [63], GAP31 from* Gelonium multiflorum* [64], and jacalin [65] are inhibitory on viral proliferation, including HIV and cytomegalovirus by potentially inhibiting viral interaction with critical host cell components. Due to the versatile antifungal, antibacterial, and antiviral functions delivered by these compounds, it is advantageous to investigate in depth their exact mechanism of action.

## 3. Critical Antimicrobial Properties of PDAs

### 3.1. Membrane Disruption and Impaired Cellular Metabolism

Although the exact mechanisms by which PDAs exert their antimicrobial action are not well defined, several potential methods have been reported. These include disruption of bacterial cell membrane leading to loss of membrane potential, impaired ATP production, and leakage of intracellular contents [66, 67]. Furthermore, chelation of metal ions, inhibition of membrane-bound ATPase, and altered membrane permeability brought about by PDAs affect normal physiology of bacteria and cause cell death [12, 32, 34, 68–71]. Plant-derived antimicrobials such as carvacrol, thymol, eugenol, and catechins act by disruption of cell membrane, followed by the release of cell contents and loss of ATP [12, 70, 72, 73]. However, cinnamaldehyde has been reported to result in the depletion of intracellular ATP by inhibiting ATPase dependent energy metabolism along with the inhibition of glucose uptake and utilization [32, 69, 70, 74]. Lysis of cell wall has also been documented in bacteria exposed to phenolic compounds [32, 75].

### 3.2. Antibiofilm Activity

Bacterial biofilms are surface-associated microbial communities enclosed in a self-generated exopolysaccharide matrix [76, 77]. They are a cause of major concern, especially in the food industry and hospital environments due to their recalcitrance to commonly used antimicrobials and disinfectants [78–82], thereby resulting in human illnesses, including endocarditis, cystic fibrosis, and indwelling device-mediated infections [83].

Extensive research exploring the potential of alternative strategies for microbial biofilm control has highlighted the efficacy of several PDAs in controlling biofilm formation in major pathogens, including* Listeria monocytogenes* [84],* Staphylococcus aureus* [85–89],* Pseudomonas aeruginosa* [90, 91],* Escherichia coli* [92, 93], and* Klebsiella pneumoniae* [94].* Trans*-cinnamaldehyde, an aromatic aldehyde obtained from bark of cinnamon trees, was found to inhibit biofilm formation and inactivate mature biofilm of* Cronobacter sakazakii* on feeding bottle coupons, stainless steel surfaces, and uropathogenic* E. coli* on urinary catheters [95, 96]. Similarly, terpenes such as carvacrol, thymol, and geraniol and essential oils of* Cymbopogon citratus* and* Syzygium aromaticum* were found to exhibit marked antibiofilm activity against both fungal [97–99] and bacterial biofilms [86, 87, 100] encountered in food processing environments and biomedical settings.

As observed in antibiotics [101–103], PDAs at subinhibitory concentrations (SICs, concentrations not inhibiting the growth of microbes) are reported to modulate bacterial gene transcription [84, 96, 104–106], which could be a contributing factor to their antibiofilm property. In a study by Amalaradjou and Venkitanarayanan [96],* trans*-cinnamaldehyde was found to modulate the transcription of genes critical for biofilm formation, motility, attachment, and quorum sensing in* C. sakazakii*. Similarly, Brackman and coworkers [107] observed the inhibitory effects of* trans*-cinnamaldehyde on biofilms of* Vibrio* spp. These authors found that* trans*-cinnamaldehyde was able to mitigate autoinducer 2 based quorum sensing and biofilm formation without inhibiting bacterial growth, probably due to its effect on gene transcription. Similar transcription modulatory effects have been observed in other major pathogens such as* Salmonella* [108] and* P. aeruginosa* [109] following exposure to PDAs. Since quorum sensing is one of the key processes involved in cell-to-cell communication and social behavior in microbes, the aforementioned reports could provide new insights into the development of novel therapeutics targeting key physiological processes in microbes.

Despite exhibiting effective antibiofilm properties, the use of PDAs has been thwarted by various confounding factors such as the requirement for more contact time, difficulty in administration, and organoleptic considerations when used on food contact surfaces. Therefore several researchers have investigated the efficacy of new delivery methods such as biodegradable polymers, micellar encapsulation, and polymeric films to potentiate the antibiofilm action of plant compounds. For example, micellar encapsulated eugenol and carvacrol were found to inhibit and inactivate* L. monocytogenes* and* E. coli* O157:H7 colony biofilms [110]. Similarly, reduced biofilm formation was observed on polymeric films containing carvacrol and cinnamaldehyde [88]. Nanoparticle-based drug delivery systems have been more frequently investigated for potentiating the antimicrobial efficacies of drugs [111]. The major advantages of nanoparticle-based drug delivery include sustained release, higher stability, and enhanced interaction of active ingredients with pathogens at their molecular level [112], thereby potentiating their antimicrobial action. The antimicrobial potential of nanoparticles containing plant-derived compounds such as* trans*-cinnamaldehyde, eugenol [113], and resveratrol [114] or essential oil of* Nigella sativa* [115] and garlic [116] has been recently investigated. These researchers found that nanoparticle formulations were more stable and highly effective in inhibiting the growth of major bacterial pathogens, including* Salmonella* and* Listeria* spp. Currently research is underway to investigate the potential of various nanoparticle-based delivery systems containing PDAs [117] for eradicating biofilms from hospital devices [118] and food processing environments [119]. In a recent study, Iannitelli and coworkers [117] prepared carvacrol encapsulated poly (DL-lactide-co-glycolide) (PLGA) nanoparticles and found that they were significantly effective in inactivating microbial biofilms of* Staphylococcus epidermidis.* In another study, PLGA containing cinnamaldehyde and carvacrol coatings were found to inhibit biofilms of* E. coli*,* S. aureus*, and* P. aeruginosa* [120].

### 3.3. Inhibiting Bacterial Capsule Production

Polysaccharide capsule is an important virulence determinant [121, 122] in many pathogenic bacteria, including* Streptococcus pneumonia* [123–125],* S. aureus* [126],* K. pneumoniae* [127], and* Bacillus anthracis* [128]. It protects bacteria from phagocytosis [123], thereby enhancing bacterial survival inside the host [126]. In addition, the presence of a capsule enhances bacterial adhesion and biofilm formation [129] in the environment [10, 130]. Bacterial capsule has also been observed to cause pathology in plants. For example, capsular polysaccharide of* Pseudomonas solanacearum* was found to occlude xylem vessels resulting in plant death [131]. Since salicylic acid is a signal molecule involved in plant defense [132], several researchers have investigated the effect of salicylic acid [133] or its derivatives such as sodium salicylate [134], bismuth subsalicylate [135], and bismuth dimercaprol [136] on modulating bacterial capsule production. These researchers found that salicylic acid or its derivatives were effective in significantly reducing capsule production by modulating the expression of global regulators controlling capsular synthesis in* S. aureus*. Similar inhibitory effects have been observed with sub-MICs and MICs of various antibiotics [137–140]. Thus, plant-derived compounds represent a valuable resource for the development of therapeutics targeting bacterial capsule production.

### 3.4. Increasing Antibiotic Susceptibility in Drug Resistant Bacteria

As the understanding of antimicrobial resistance mechanisms in pathogens is increasing, multifold strategies to combat infections and reverse bacterial antibiotic resistance are being explored. Many researchers have reported PDAs as potential resistance modulating compounds, in addition to their inherent antimicrobial nature. In a study by Brehm-Stecher and Johnson [141], low concentrations of sesquiterpene such as nerolidol, bisabolol, and apritone increased bacterial sensitivity to multiple antibiotics, including ciprofloxacin, clindamycin, tetracycline, and vancomycin. Similarly, Dickson et al. [142] reported that plant extracts from* Mezoneuron benthamianum*,* Securinega virosa*, and* Microglossa pyrifolia* increased the susceptibility of major drug resistant fungi such as* Trichophyton* spp. and* Microsporum gypseum* and bacteria such as* Salmonella* spp.,* Klebsiella* spp.,* P. aeruginosa*, and* S. aureus* to norfloxacin. In addition, geraniol (present in essential oil of* Helichrysum italicum*) was found to restore the efficacy of quinolones, chloramphenicol, and *β*-lactams against multidrug resistant pathogens, including* Acinetobacter baumannii* [143]. Similar synergism was observed between antibiotics and various other medicinal plant extracts, including those of* Camellia sinensis* [144],* Caesalpinia spinosa* [145], oil of* Croton zehntneri* [146], carvacrol [147], and baicalein, the active component derived from* Scutellaria baicalensis* [148]. This modulatory effect of plant compounds is potentially due to the attenuation of three main resistance strategies employed by drug resistant pathogens to survive the action of antibiotics, namely, enzymatic degradation of antibiotics [149], alteration of antibiotic target site [150], and efflux pumps [151]. In addition, recent reports suggest that the combination therapy of antibiotics with PDAs acts through inhibition of multiple targets in various pathways critical for the normal functioning or virulence of the bacterial cell.

Generation of *β*-lactamase enzymes is an example of microbial strategy that is responsible for resistance to *β*-lactam antibiotics [152]. Several plant compounds have been identified with inhibitory activity towards *β*-lactamases [153]. Gangoué-Piéboji and coworkers [154] screened medicinal plants from Cameroon and found that extracts from* Garcinia lucida* and* Bridelia micrantha* exhibited significant inhibitory activity towards *β*-lactamases. Similarly, epigallocatechin gallate was found to inhibit penicillinase activity, thus increasing the sensitivity of* S. aureus* to penicillin [155] and augmenting the antimicrobial properties of ampicillin and sulbactam against Methicillin resistant* S. aureus* (MRSA).

Numerous studies in the past two decades have shown the efficacy of PDAs as potent efflux pump inhibitors against Gram-positive microbes [156–158]. Gram-negative bacteria pose an even greater challenge owing to the presence of potent efflux pumps, especially, AcrAB-TolC pumps [159]. In a recent investigation, five PDAs, namely,* trans*-cinnamaldehyde, *β*-resorcylic acid, carvacrol, thymol, and eugenol, or their combinations were found to increase the sensitivity of* Salmonella enterica* serotype Typhimurium phage type DT104 to five antibiotics [160]. Since the mechanism of antimicrobial resistance in* Salmonella* Typhimurium DT104 is mainly mediated by interaction between specific transporters of antibiotics and AcrAB-TolC efflux pump, the aforementioned plant compounds could be acting through modulation of these efflux pumps to increase the antibiotic sensitivity of the pathogen [161].

### 3.5. Attenuating Bacterial Virulence

The pathophysiology of microbial infection in a host is mediated by multiple virulence factors, which are expressed at different stages of infection to cause the disease. Reducing production of these virulence factors could control infections in humans. With major advancement in the fields of comparative genomics, transcriptomics, and proteomics, a better understanding of the key virulence mechanisms of pathogenic bacteria has been achieved. Thus, virulence factors are the prime targets for therapeutic interventions and vaccine development [11]. Quorum sensing controls the expression of genes encoding various virulence factors in many microorganisms [162, 163]. A growing body of evidence suggests that plants produce antiquorum sensing compounds that interfere with cell-to-cell communication, thereby downregulating the expression of virulence genes in microbes [164–166]. We previously reported that* trans*-cinnamaldehyde reduced the expression of* luxR*, which codes for transcriptional regulator of quorum sensing in* C. sakazakii* [96]. Similarly, Bodini and coworkers found that garlic extract and p-coumaric acid inhibited quorum sensing in quorum sensing reporter strains, indicating that plant compounds potentially modulate virulence by affecting quorum sensing in microbes.

For the majority of enteric pathogens, adhesion to and invasion of intestinal epithelium are critical for virulence and infection in a host. Specific proteins contribute to adhesion and invasion in various microbes. For example, Inl A and Inl B are surface proteins that facilitate receptor-mediated entry of* L. monocytogenes* in intestinal cells [167]. Several PDAs have been shown to reduce these virulence attributes in major food-borne pathogens such as* L. monocytogenes* [105], uropathogenic* E. coli* [168], and* Salmonella enterica* serovar Enteritidis [104] by downregulating the expression of virulence genes. In addition, reduction in capsule production has been documented in* K. pneumoniae* on exposure to PDAs [169], which affects its virulence and survival inside the host. These results highlight the ability of plant compounds to successfully target virulence factors critical for pathogenicity and pave the way for the development of compounds that target bacterial virulence.

### 3.6. Reducing Toxin Production

Microbial toxins are chemical compounds critical for virulence and pathogenesis in the host and therefore are prime targets for therapeutic interventions. Microbial toxins include exotoxins (secreted by the bacteria) and endotoxins (released after bacterial lysis), whereas mycotoxins are toxic secondary metabolites produced by fungi with diverse chemical structures and biological activities causing a variety of illnesses in humans. The drugs of choice for treating bacterial infections have been antibiotics; however the use of antibiotics to kill toxigenic microorganisms has several disadvantages such as resistance development [170], disruption of normal microbiota [171], and enhanced pathogenesis due to increased toxin production and cell lysis as observed in* E. coli* O157:H7 [172, 173]. Moreover, toxin-mediated pathogenesis can continue in the host even after bacterial clearance [174]. Therefore, antibiotics in general are contraindicated to treat toxigenic organisms and it is beneficial to employ an alternative approach to counteract the toxin-mediated virulence of pathogens.

In the past, plant extracts and their active molecules have proven effective against bacterial toxins produced by* Vibrio* spp.,* S. aureus*,* E. coli*, and fungal toxins from* Aspergillus* spp. For example, a natural plant-derived dihydroisosteviol has been observed to prevent cholera toxin-mediated intestinal fluid secretion [175]. Plant polyphenols such as RG-tannin and apple phenols have been reported to inhibit ADP-ribosyltransferase activity critical for cholera toxin action [176, 177]. These researchers also observed a reduction in the toxin induced fluid accumulation in mouse ileal loops. In a recent study by Yamasaki et al. [178], extracts from spices such as red chilli, sweet fennel, and white pepper were found to substantially inhibit the production of cholera toxin. These researchers found that capsaicin was an important component among the tested fractions and significantly reduced the expression of major virulence genes of* V. cholerae*, including* ctxA*,* tcpA*, and* toxT*. Similarly, eugenol, an essential oil from clove, was observed to significantly reduce the production of* S. aureus* *α*-hemolysin, enterotoxins (SEA, SEB), and toxic shock syndrome toxin 1 [106]. Transcriptional analysis conducted by these researchers revealed a reduction in the expression of critical virulence genes (*sea, seb, tst, and hla*) involved in various aspects of* S. aureus* toxin production. Similarly, a compound from olive, 4-hydroxytyrosol, was found to successfully inactivate* S. aureus* endotoxin production* in vitro* [179].

Enterohemorrhagic* E. coli* (EHEC) is responsible for causing severe human infections, characterized by hemorrhagic colitis and hemorrhagic uremic syndrome [180]. In a recent study by Doughari and coworkers [181], extracts of* Curtisia dentata* were found to inhibit expression of* vtx1* and* vtx2* genes in EHEC. The extracts from this plant have been traditionally used as an antidiarrheal agent [182]. Similar verotoxin inhibitory activity was observed in other plant extracts such as* Haematoxylon brasiletto* [183],* Limonium californicum* (Boiss.),* Cupressus lusitanica*,* Salvia urica* Epling, and* Jussiaea peruviana* L. [184]. Inactivation of Shiga toxins by antitoxin antibodies [185] and by certain synthetic carbohydrate and peptide compounds designed to compete with the active site of the toxin for receptor sites on cell membranes has also been investigated [186–189]. Quiñones and coworkers [190] found that grape seed and grape pomace extracts exhibited strong anti-Shiga toxin-2 activity and conferred cellular protection against Shiga toxin-2. Likewise, Daio (*Rhei rhizoma*), apple, hop bract, and green tea extracts have been shown previously to inhibit the release of Shiga toxin from* E. coli* O157:H7 [176, 191].

Aflatoxins, produced by* Aspergillus flavus, A. parasiticus, A. nomius, A. tamari, A. bombycis, *and* A. pseudotamarii*, cause both acute and chronic toxicity in humans and animals [192–195]. Common food products associated with mycotoxicosis include peanuts, corn grain, cottonseed [196, 197], chicken meat [198] cheese [199], canned mushrooms [200], raw milk [201, 202], and pork [203, 204]. Several studies have highlighted the efficacy of essential oils in reducing mycotoxin production. Crude aqueous extracts of garlic, carrot, and clove have been shown to exert a significant inhibitory effect on aflatoxin production in rice [205]. Capsanthin and capsaicin, the coloring and pungent ingredients of red chilli (*Capsicum annum*), completely inhibited both the growth and toxin production in* A. flavus* [206]. Mahmoud [207] studied the effect of several plant essential oils on growth and toxin production of* A. flavus* and found that five essential oils, namely, geraniol, nerol, citronellol, cinnamaldehyde, and thymol, completely suppressed the growth of* A. flavus* and prevented aflatoxin synthesis in a liquid medium. Similarly, curcumin and essential oil from* Curcuma longa* have also been reported to inhibit* A. flavus* toxin production [208]. In another study, cumin and clove oils have been found to exert inhibitory effects on toxin production in* A. parasiticus* [209], wherein aflatoxin production was decreased by 99%. Similar findings have been observed with ochratoxin-producing aspergilli, where essential oil from wild thyme reduced ochratoxin production by more than 60% [210]. In addition, essential oils have been found to inhibit spore germination in toxin producing* Aspergillus* species [211]. In a recent study, Kumar and coworkers [212] demonstrated that amaryllin, a 15-kDa antifungal protein from* Amaryllis belladonna* bulbs, exerts significant inhibitory effect against toxin producing* A. flavus* and* Fusarium oxysporum*. The aforementioned studies collectively suggest that plant polyphenols and other plant compounds are potential agents that can be used to protect humans against toxin-mediated food-borne diseases.

### 3.7. Beneficial Effects on Host Immune System

Pioneering research has demonstrated the existence of intriguing parallels between plant and animal immune responses against microbial infections. These include recognition of invariant pathogen-associated molecular patterns (PAMPs) [213], apoptosis of infected cells [214, 215], and production of antimicrobial peptides [216, 217]. However, unlike microbe-specific immune response in animals, plants depend on innate immunity of individual cells coupled with signals emanating from the site of infection [28, 218–220] to combat infections. This is mediated by the production of a wide variety of low molecular weight secondary metabolites [26, 221]. A mounting body of evidence suggests that plants extracts, in addition to their role in plant defense, exert immune-modulatory effects in animals [222, 223] and are increasingly being used for treating inflammatory diseases, allergy, and arthritis [224]. For example, tea tree [225, 226] and lavender oils [227] were found to ameliorate allergy symptoms by reducing histamine release [228, 229] and cytokine production [230]. The immune-modulatory effects of many PDAs have been demonstrated in mouse, chicken, and human cell lines [231–233]. Since the majority of the enteric pathogens colonize and invade the gut epithelium, followed by systemic spread via macrophages resulting in infection, the intestinal mucosal immune response (IMIS) is critical for conferring protection against such bacterial infections. A growing body of evidence suggests that PDAs in addition to attenuating bacterial virulence modulate IMIS [224, 234] through both nonspecific inflammatory response and antigen specific adaptive interactions in the intestine, thereby affecting pathogen survival. Plant preparations such as* Eucalyptus* oil [224], babassu mesocarp extract [235], and oil from seeds of* Chenopodium ambrosioides *L. [236] were found to activate the phagocytic activity of macrophages, whereas essential oils from* Petroselinum crispum* [234],* Artemisia iwayomogi* [237], and Jeju plant extract [116] were found to suppress activity of splenocytes and macrophages, indicating that the two oils may act through different mechanisms.

### 3.8. Beneficial Effects on Gastrointestinal Microflora

The human intestinal tract hosts a vast population of diverse bacterial communities that amount to as many as 10^12^ cells per 1 g of fecal mass in an average human being [238, 239]. The gut microbiota interacts with the host and influences various biological processes [240], including microbial defense [241]. With advances in high throughput sequencing and metagenomics and development of gnotobiotic animals, the ability to explore the variations in gut microbiota composition and their effect on human health has significantly improved [242, 243]. Modulations in dietary components have been associated with fluctuations in the composition of gut microbial population and diversity [244, 245], which in turn affects host's metabolic functions [246] and susceptibility to gastrointestinal bacterial infections [247]. David and coworkers [248] observed that short-term macronutrient variation leads to a change in the gut microbial community structure, with animal protein-based diet increasing the abundance of bile-tolerant microorganisms (*Alistipes*,* Bilophila*, and* Bacteroides*) and reducing the levels of Firmicutes that metabolize dietary plant polysaccharides (*Roseburia*,* Eubacterium rectale*, and* Ruminococcus bromii*). Bailey and group [249] demonstrated that stress exposure disrupted commensal microbial populations in the intestine of mice and led to increased colonization of* Citrobacter rodentium*. These researchers in their subsequent study observed that* Lactobacillus reuteri* attenuated the stress-enhanced severity of* C. rodentium* infection in mice [250]. Interestingly, recent studies have shown that PDAs that are highly bactericidal towards enteric pathogens exert low antimicrobial effect against commensal gut microbiota [251, 252]. Thapa and coworkers [253] found that nerolidol, thymol, eugenol, and geraniol inhibited growth of enteric pathogens such as* E. coli* O157:H7,* Clostridium difficile*, and* S. Enteritidis*. Moreover, the degree of inhibition was more on the pathogens than the commensal bacteria. Since PDAs and probiotics exert their antimicrobial effects by different mechanisms [254], a combinatorial approach using both could be more effective in controlling pathogens as compared to using them separately. However, research investigating their synergistic interactions is scanty. Further research is necessary to comprehensively elucidate the mechanism of action of such dietary interventions and their effect on gut microbiota for designing effective therapies that promote health by targeting diverse microbial communities.

## 4. Challenges Associated with Using PDAs for Pathogen Control

The efficacy of PDAs in controlling pathogens in the environment, high-risk foods, or their virulence in the host depends on various intrinsic and extrinsic factors. Physiochemical properties of PDAs such as solubility in aqueous solutions, hydrophobicity, biodegradability, and stabilities are major challenges that thwart their usage as natural biocontrol agents in the environment [32, 255]. In addition, factors such as environmental temperature and atmospheric composition also modulate their antimicrobial efficacy [256]. In food products, the presence of fat [257], carbohydrates [258], and proteins [259] affects the efficacy of PDAs. Moreover, chemical variability in PDAs, originating from differences in extraction protocols [260, 261], affects the antimicrobial efficacy [12]. Another concern for PDAs is their strong aroma, which may modulate the organoleptic property and taste profile of food products. Therefore, careful selection of PDAs based on their chemical composition and effect on sensory attributes of food product is warranted before recommending their usage as food preservatives or direct oral supplements for human consumption [262].

## 5. Future Directions

With an increasing body of supporting literature, PDAs are now recognized to play a critical role in the development of effective therapeutics, either alone or in combination with conventional antibiotics. However, the major challenges to this include finding compounds with sufficiently lower MICs, low toxicity, and high bioavailability for effective and safe use in humans and animals.

Based on their modes of action, PDAs are classified into three categories, including conventional antimicrobials, multidrug resistance inhibitors, and compounds that target specific or multiple virulence factors in microbes [221]. As new approaches that target specific regulatory pathways and bacterial virulence are becoming the paradigm of antibacterial therapeutics in recent years, characterization of the mechanism of action of these compounds would pave the way for the development of novel drugs that can circumvent antimicrobial resistance and control infectious diseases.

## Figures and Tables

**Table 1:** Chemical structure, examples, and antimicrobial spectrum of major groups of plant-derived antimicrobials.

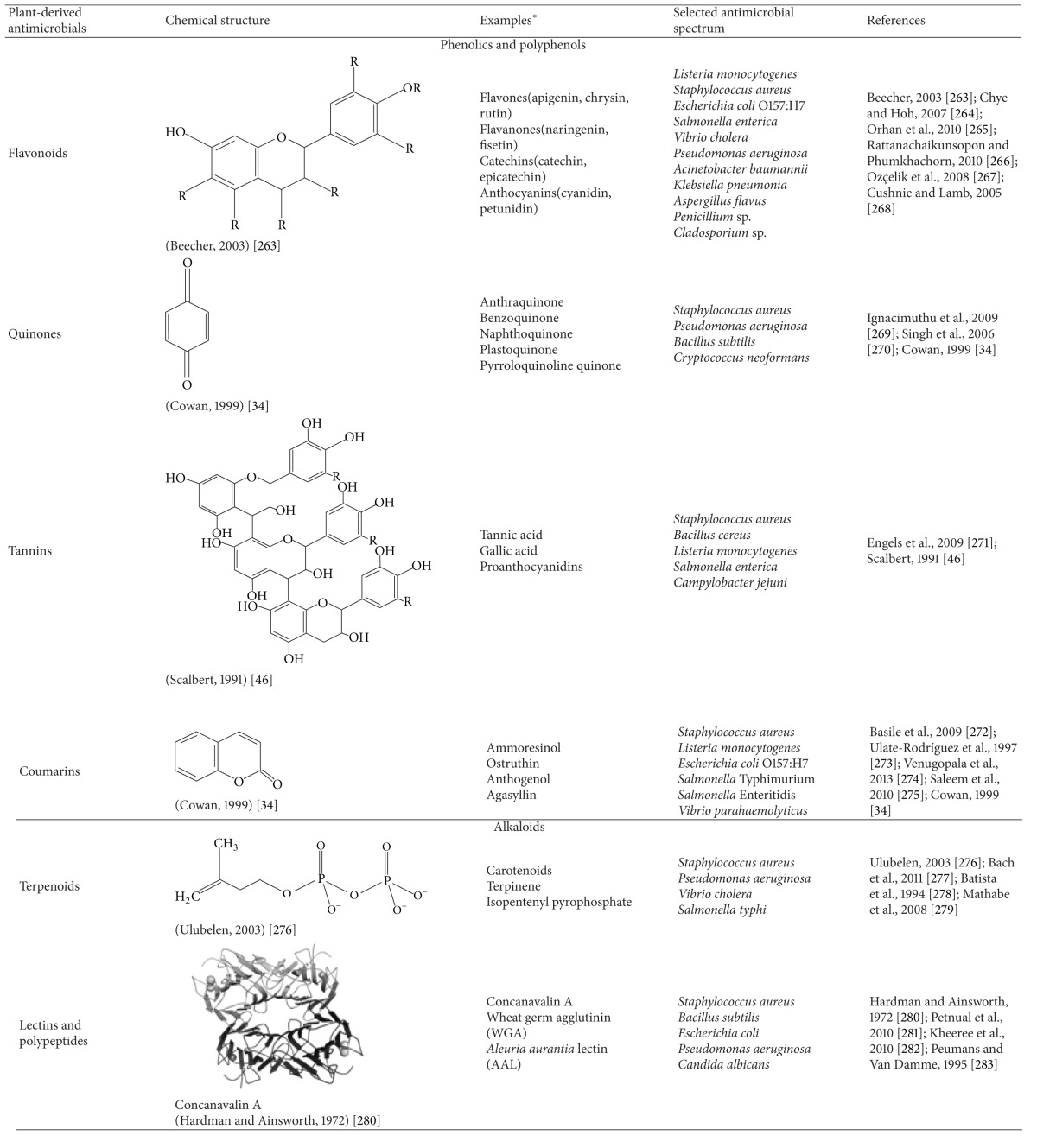

*The examples discussed in the table are only representative for the group. For an extended list of examples of each group, the readers are requested to peruse review articles in the References section and other sources.
